# Fate-tox: fragment attention transformer for E(3)-equivariant multi-organ toxicity prediction

**DOI:** 10.1186/s13321-025-01012-5

**Published:** 2025-05-14

**Authors:** Sumin Ha, Dongmin Bang, Sun Kim

**Affiliations:** 1 Interdisciplinary Program in Artificial Intelligence, Seoul National University, Seoul, 08826 Republic of Korea; 2 Interdisciplinary Program in Bioinformatics, Seoul National University, Seoul, 08826 Republic of Korea; 3 AIGENDRUG Co., Ltd., Seoul, 08758 Republic of Korea; 4 Department of Computer Science and Engineering, Seoul National University, Seoul, 08826 Republic of Korea

**Keywords:** Transformer, Graph neural network, Toxicity prediction, Multi-task learning

## Abstract

Toxicity is a critical hurdle in drug development, often causing the late-stage failure of promising compounds. Existing computational prediction models often focus on single-organ toxicity. However, avoiding toxicity of an organ, such as reducing gastrointestinal side effects, may inadvertently lead to toxicity in another organ, as seen in the real case of rofecoxib, which was withdrawn due to increased cardiovascular risks. Thus, simultaneous prediction of multi-organ toxicity is a desirable but challenging task. The main challenges are (1) the variability of substructures that contribute to toxicity of different organs, (2) insufficient power of molecular representations in diverse perspectives, and (3) explainability of prediction results especially in terms of substructures or potential toxicophores. To address these challenges with multiple strategies, we developed FATE-Tox, a novel multi-view deep learning framework for multi-organ toxicity prediction. For variability of substructures, we used three fragmentation methods such as BRICS, Bemis-Murcko scaffolds, and RDKit Functional Groups to formulate fragment-level graphs so that diverse substructures can be used to identify toxicity for different organs. For insufficient power of molecular representations, we used molecular representations in both 2D and 3D perspectives. For explainability, our fragment attention transformer identifies potential 3D toxicophores using attention coefficients.

**Scientific contribution**: Our framework achieved significant improvements in prediction performance, with up to 3.01% gains over prior baseline methods on toxicity benchmark datasets from MoleculeNet (BBBP, SIDER, ClinTox) and TDC (DILI, Skin Reaction, Carcinogens, and hERG), while the multi-task learning approach further enhanced performance by up to 1.44% compared to the single-task learning framework that had already surpassed these baselines. Additionally, attention visualization aligning with literature contributes to greater transparency in predictive modeling. Our approach has the potential to provide scientists and clinicians with a more interpretable and clinically meaningful tool to assess systemic toxicity, ultimately supporting safer and more informed drug development processes.

## Introduction

Drug development remains hindered by the critical challenge of toxicity, which often leads to drug failures [1, 2]. Consequently, early and accurate identification of potentially harmful compounds is pivotal in drug development, which led to development of machine-learning based predictors of toxicity [3, 4]. Despite advances in deep learning-based modeling of compounds, existing models predominantly focus on predicting toxicity for a single organ [5–8], neglecting the systemic nature of chemical toxicity [9].

The human body is an integrated system where organs interact through complex biochemical and physiological pathways. Several drugs have been withdrawn from the market due to unforeseen systemic toxicity, despite passing initial organ-specific safety tests. For example, Troglitazone was withdrawn due to liver toxicity, but is also revealed to be associated with cardiovascular risks [10]. Rofecoxib was withdrawn due to increased risk of heart attack and stroke, despite initial focus on gastrointestinal safety [11].

This interconnectedness underscores the importance of simultaneous multi-organ toxicity prediction in drug discovery. Single-organ toxicity models fail to account for the interconnectedness of organ systems and secondary toxic effects, which is essential for understanding systemic toxicity [9]. Hence, a comprehensive modeling of chemical toxicity across multiple organs is essential to mitigate risks and ensure the safety of drugs. While graph-based deep learning models have shown promise in predicting various toxicity endpoints [5–8], they face three significant hurdles when applied to multi-organ toxicity prediction.

The first challenge for the whole-molecule scale representation is to effectively capture the intricate and multi-view characteristics inherent in molecular structures. Both 2D topological information and 3D spatial arrangements play crucial roles in molecular interactions, and integrating these perspectives can provide a more comprehensive understanding. While 3D conformation is essential for modeling interactions such as drug binding, the 2D topology remains fundamental in defining key molecular properties, including toxicity [12]. Therefore, a multi-view approach that incorporates both 2D and 3D representations is required for more robust molecular modeling. 

Building upon this, the second key challenge is identifying diverse subgraphs that contribute to varying toxicity endpoints through distinct mechanisms. The subgraph scale is critical as it elucidates more about organ-specific biochemical pathways and physiological mechanisms, such as liver cytochrome P450 enzyme activity or kidney tubular transport systems, which demands more fine-grained substructure features [13]. However, there is no single well-defined substructure sufficient to explain all toxic effects. For example, the PAINS filter employs multiple criteria to flag substructures associated with colloidal aggregation, redox activity, covalent reactivity and chelation which are all critical contributors to distinct toxicities like hepatotoxicity, genotoxicity or immunotoxicity [14]. Such variations in toxicophores and its mechanisms make it difficult to build generalizable models that reliably predict toxicity across different molecules and organs. 

Lastly, the explainability of toxicity prediction, particularly in identifying contributing substructures within the context of 3D molecular space, remains largely unexplored in existing works. Recent toxicity prediction methods [15–17], are designed to provide explainability through structural alerts (SAs), utilizing SHapley Additive exPlanations (SHAP) analysis on 2D graph-based molecular fingerprints.

In order to address these challenges through a holistic and interpretable approach for multi-organ toxicity prediction, we propose FATE-Tox (Fragment Attention Transformer for E(3)-Equivariant Toxicity Prediction), a novel framework that integrates structural modeling and interpretive capabilities. FATE-Tox uniquely combines a transformer-based stage for computing invariant features further processed with E(3)-equivariant graph neural networks, enabling robust and accurate 3D-aware molecular modeling. This multi-view approach captures structural and interaction patterns of molecules that contribute to systemic toxicity, overcoming the first hurdle in multi-organ toxicity. Furthermore, it incorporates fragment-level graphs generated through three distinct fragmentation methods - BRICS, Bemis-Murcko scaffolds and RDKit Functional Groups - providing a multigranular view of molecular structures based on chemical knowledge accounting for metabolic and degradation pathways throughout the human body. The variation in subgraphs provided to the dual-branch architecture, which aligns atom- and fragment-level representations, enhances the model’s ability to generalize effectively across diverse toxicity endpoints. 

To our knowledge, FATE-Tox is the first framework to achieve a comprehensive prediction of toxicity, utilizing 3D molecular information. Unlike prior models that lack interpretability, our framework offers explainable insights into the toxicophores responsible for toxicity by incorporating attention visualization. This approach provides a level of transparency that is rarely achieved in graph-based models, enabling a clearer understanding of how specific molecular substructures contribute to toxicity. 

FATE-Tox achieves state-of-the-art results on benchmark toxicity datasets and demonstrates robust multi-task learning across diverse endpoints, validating its effectiveness in addressing the systemic nature of chemical toxicity. Comprehensive case studies on attention coefficient visualization and embedding space analysis highlight our model’s interpretability and the effectiveness of its simultaneous multi-organ toxicity prediction strategy, especially by leveraging multiple fragmentation methods.

## Related work

### Molecular property prediction

Molecular representation learning (MRL) has gained significant attention in recent years due to its potential in drug discovery, material science, and other chemical applications. The field has seen diverse approaches based on the input format of molecular data, from molecular fingerprints and 1D SMILES strings to 2D molecular graphs and 3D conformers.

**2D topological graph-based models**. Prior to the development of graph-based deep learning modeling, quantitative-structure activity/toxicity relationships (QSAR/QSTR) approaches were widely explored [18]. Speck-Planche et al. [19] introduced multi-target QSAR (mtk-QSAR), leveraging Artificial Neural Networks and cheminformatics-derived molecular descriptors to predict multiple toxicity endpoints [20]. These models, also known as Perturbation-Theory Machine Learning (PTML), are advanced 2D-QSAR methods that integrate chemical and biological information across various complexity levels. PTML enables simultaneous prediction of multiple endpoints across diverse biological targets (e.g., proteins, microbes, cell lines) and assay protocols [21].

With the advent of graph-based deep learning architectures, development of MRL models have shifted towards 2D molecular graphs, which represent atoms as nodes and bonds as edges. These models aim to learn molecular properties through graph neural networks (GNNs) by capturing the topological structure of molecules. MolCLR [22] utilizes data augmentation at both the node and graph levels and employs a contrastive learning strategy. GraphMVP [23] introduces a contrastive learning framework between 2D topology and 3D molecular geometry, aiming to bridge these two representations for better alignment. With the emergence of Transformers [24] and their applications in graph data, Molecular Attention Transformer [25, 26] attempts to alter the attention mechanism suited to comprehend molecular graphs.

**3D conformer-based models**. To further enhance molecular understanding, recent models [27, 28] have extended to incorporate 3D conformers, capturing the spatial arrangement of atoms. However, ensuring that models account for transformations in 3D space, such as rotations and translations, is crucial to maintain consistent and reliable predictions. For this reason, the E(3) equivariance is often necessary in molecule property prediction tasks. It guarantees that the model’s output remains invariant when the input undergoes these transformations, which is vital for learning physical properties that are inherently symmetrical. A variety of E(3)-equivariant models have been developed for supervised learning tasks [29–32] involving energy and force predictions.

By leveraging various molecular representations-1D, 2D, and 3D-combined with the development of E(3)-equivariant architectures, modern MRL frameworks are well-equipped to tackle a range of challenges in the molecular domain.

### Organ-specific toxicity prediction models

The computational modeling of toxicity has evolved to address the challenges of efficiently assessing chemical hazards. Traditionally, most toxicity prediction models have been developed to focus on specific endpoints, such as hepatotoxicity [8], cardiotoxicity [5, 33], or carcinogenicity [6]. These endpoint-specific models [7] are tailored to particular biological mechanisms and datasets, allowing them to achieve high accuracy within narrow domains. Several models have been proposed to further provide interpretability regarding the toxicity of compounds. CardioDPi [15] and RespirationDPi [16] are explainable deep learning models for predicting cardiotoxicity and respiratory toxicity, respectively, along with explainability using Structure Alerts (SA) and SHAP analysis. Furthermore, BCDPi [17] adopted a multi-task learning framework to predict environmental toxicity based on chemical bioconcentration in fish, along with interpretability of substructures using SHAP analysis based on the Klekota-Roth fingerprint (KRFP).

However, their specialized nature limits their generalizability, making it difficult to predict comprehensive toxicity profiles that span multiple biological systems.

In contrast, attempts at comprehensive toxicity prediction have traditionally relied on simplified molecular representations such as fingerprints-binary or numerical vectors encoding the presence or absence of predefined substructures [34–37]. These fingerprints, when combined with machine learning models like Random Forests or Support Vector Machines, offer a straightforward approach to assessing multiple toxicity endpoints simultaneously. While efficient, this approach suffers from limited expressiveness, as fingerprints often fail to capture subtle structural and stereochemical features critical for accurate predictions. Consequently, such models may struggle to distinguish between structurally similar compounds or enantiomers with differing toxicity profiles.

Emerging approaches integrating advanced molecular representations, including graph-based [38, 39] and 3D-aware methods [40], seek to address these limitations.

These newer methods hold promise for achieving comprehensive toxicity prediction with greater accuracy and interpretability.

## Methods

### Datasets

In order to demonstrate the utility of our model on prediction of toxicity, we conducted experiments on a wide range of toxicity-related datasets sourced from MoleculeNet [41] and the Therapeutics Data Commons (TDC) [2], two well-established benchmarks in drug discovery and toxicity prediction. These datasets provide a robust foundation for toxicity prediction, encompassing specific organ toxicities (e.g., hepatotoxicity and neurotoxicity) as well as broader systemic toxicity endpoints (side effects and approval/withdrawal labels). Consistent with methodologies in previous studies, the Moleculenet datasets are divided using scaffold splitting, where test set contains unseen Murko scaffolds, in order to evaluate the generalizability of the models. For TDC datasets, we follow the scaffold-splitted subsets given in the datasets. The detailed statistics and sources of the benchmark datasets are summarized in Table 1.

**Table 1:** Benchmark toxicity datasets, their statistics and origins

Task	BBBP	SIDER	ClinTox	DILI	Skin Rxn	Carcinogens	hERG
	Binary classification						
Origin	Moleculenet			TDC			
Num. of tasks	1	27	2	1	1	1	1
Recovered	2039 (99.85%)	1427 (97.20%)	1478 (99.85%)	475 (100%)	403 (100%)	276 (100%)	643 (99.23%)
Train/val/test	1628/204/204	1109/139/139	1167/146/146	332/47/96	281/40/82	193/28/55	453/65/132

“Recovered” represents the count of molecules successfully embedded as both graph structures and 3D conformers

### FATE-Tox: fragment attention transformer with E(3)-equivariance

#### Fragmentation methods

**Fig. 1:**
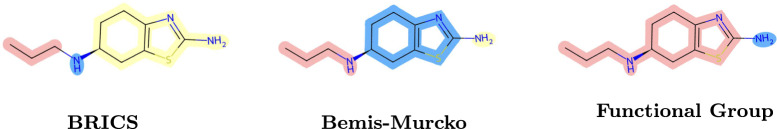
Visualization of Fragmentation Methods. Pramipexole, a dopamine agonist used to treat the symptoms of Parkinson disease, fragmentized based on the three fragmentation methods, BRICS, Murcko and Functional Group (RDKit). BRICS fragment pramipexole into smaller substructures whereas Murcko decomposes it to its core scaffold, retaining the fused bicyclic aromatic system and the thiazole ring. Functional Group-based fragmentation generates a large backbone structure, primarily focusing on the primary amine

Fragmentation methods dissect complex molecule structures into smaller fragments, offering insights into critical structural features or interactive sites (Fig. 1). Reflecting its importance in medicinal chemistry and chemical informatics, various fragmentation methods have been proposed - differing on disruption criteria (e.g., cyclic structures, double bonds) or predetermined fragment libraries [42]. Diverse fragmentation methods that capture distinct chemical aspects within a molecule can provide valuable insights for comprehensive toxicity prediction, especially considering that molecules may exist in various forms within the body due to different pH environments and metabolic processes. We employ three different fragmentation methods: BRICS, Bemis-Murcko scaffolds, RDKit functional group. The combination of these approaches enables a more exhaustive understanding of structure-activity relationships in toxicity prediction.

- **BRICS (Breaking of Retrosynthetically Interesting Chemical Substructures)**. BRICS is a fragmentation method specifically designed to mimic retrosynthetic analysis [43], focusing on bonds that are commonly formed or broken in synthetic chemistry. BRICS employs 16 cleavage rules that consider the chemical environment of each bond and its surrounding substructures. This approach ensures that the resulting fragments are both chemically meaningful and synthetically accessible [42].
- **Bemis-Murcko scaffolds**. Fragmentation based on Bemis-Murcko scaffolds identifies core structures and additional side chains. Fragmentation based on such scaffolds preserves the largest ring system within the molecule and highlights peripheral side chains. Such approach can be valuable for identifying common structural motifs across a series of compounds [44].
- **Functional Groups (RDKit)**. The functional group fragmentation method utilizes predefined SMARTS patterns to identify common functional groups within molecules. We refer to the 33 functional groups defined by RDKit [45] following previous works [46]. The isolation of chemically intuitive and well-defined substructures, allows the observation of key molecular substructure’s contribution to a molecule’s overall properties and biological activity.

#### Fragment graph construction

In our representation, we model the molecule as graph $G = (V,X,A^\text {bond})$ where $V = \{1,..., N\}$ is the set of atoms in the molecule, and $X = \{(h_i, c_i^{\text {2D}}, c_i^{\text {3D}})\}_{i=1}^N$ denotes the node attributes. Each atom $i \in V$ is associated with a feature vector $h_i \in \mathbb {R}^{nf}$, 2D coordinates $c_i^{\text {2D}} \in \mathbb {R}^2$, and 3D coordinates $c_i^{\text {3D}} \in \mathbb {R}^3$. Here, *nf* is the dimensionality of the atom-level feature vector, encoding chemical properties of each atom (detailed in Appendix). The bond adjacency matrix $A^\text {bond} \in \{0,1\}^{N \times N}$ represents the molecular bonds, where $a^\text {bond}_{ij} = 1$ if atoms *i* and *j* are bonded regardless of the bond type, and 0 otherwise.

To represent fragment-level features, we partition the molecule into substructures as defined in "Fragmentation methods" section, where each fragment *k* is defined by a set of atoms $F_k \subset \{1,..., N\}$. For each fragment *k*, we define its coordinates $c_k^{\text {frag}}$ and feature vector $h_k^{frag}$ as follows. In these definitions, $F_k$ denotes the set of atoms in the *k*-th fragment, providing a straightforward method to aggregate atom-level features into fragment-level representations.

The fragment coordinates $c_k^{\text {frag}}$ are calculated as the center of mass, using weighted average of atom 3D coordinates within fragment *k* as following:

$c_k^{\text {frag}} = \frac{\sum _{i \in F_k} m_{i} c_{i}^{\text {atom}}}{\sum _{i \in F_k} m_{i}}.$ The feature vector $h_k^\text {frag}$ is obtained by applying sum-pooling to the node features of all atoms within fragment *k*:

$$h_k^{\text {frag}} = \sum _{i \in F_k} h_{i}^{\text {atom}}.$$

#### Model architecture

**Fig. 2:**
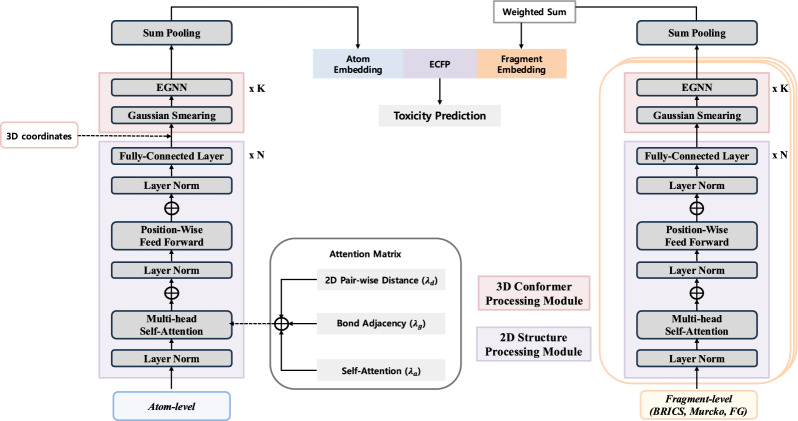
Model Architecture of FATE-Tox. The model is roughly composed of two branches: Atom-level and Fragment-level. Each branch passes down the input features and coordinates into a two-component pipeline composed of Molecular transformer module and the E(3)-Equivariant Graph Convolutional Layer. After the atom-level and aggregated fragment-level representations are generated, they are concatentated with ECFP, then passed down to the prediction MLP to yield the prediction for the toxicity label

FATE-Tox adopts a dual-branch architecture, composed of an atom-level branch and a fragment-level branch, to comprehensively capture molecular structures at different levels of granularity (Fig. 2). The atom-level branch focuses on capturing fine-grained interactions between individual atoms and their direct bonds, while the fragment-level branch guides the model to comprehend molecular structure in larger substructures, potentially understanding the concept of toxicophores. Both the atom and fragment branch is composed of a transformer module to compress 2D graph features prior to initiating message passing that incorporates 3D spatial information. The two only differ in terms of the input graph as defined in "Fragment graph construction" section.

#### Molecular transformer module

The transformer module leverages a multi-head self-attention mechanism designed to integrate molecular structural information effectively. This step aims to capture dependencies between atoms in the molecular graph, so that 2D representations with contextual information on the linkage of atom nodes, are refined and contextually enriched. Sufficient information extraction through this phase prepares molecules for subsequent message-passing operations that incorporate 3D coordinates for comprehensive molecular feature understanding.

Following the previous work [25], we integrate multi-head attention with graph structure and spatial information by incorporating adjacency and distance matrices to capture both local and global relationships within molecular structures. Given an input node feature matrix $H \in \mathbb {R}^{B \times N \times nf}$, where *B* is the batch size, *N* is the number of atoms in the molecule/fragment and *nf* is the node feature dimension, we project *H* to obtain query *Q*, key *K*, and value *V* matrices with learnable weights.

Given $n_{head}$ number of heads, the attention score matrix $P_\text {attn}$ is computed by taking the dot product between the query *Q* and key *K*, normalized by the square root of key dimension $d_k$ (where $d_k = \frac{nf}{n_{head}}$) for each head through following formula:

1 $$\begin{aligned} P_\text {attn} = \text {softmax}\left( \frac{Q_i K_j^\intercal }{\sqrt{d_k}}\right) \end{aligned}$$

for all pairs of atom *i* and *j* within a molecule.

To enhance structural understanding, the self-attention matrix is augmented with summation of bond adjacency matrix $A^\text {bond} \in \mathbb {R} ^ {B\times N \times N}$ and distance matrix $D \in \mathbb {R} ^ {B\times N \times N}$. In the molecular transformer module, the pairwise 2D distance $d_{ij}^{2D}$ between node *i* and *j* is computed using their 2D coordinates ($c_i^{2D}, c_j^{2D}$) via the RDKit Python package [45]. The bond adjacency matrix is normalized by the sum of its elements along each row to ensure proper weighting, and the distance matrix is transformed using softmax function ($D_{ij}' = \text {softmax}(-D_{ij})$) before being incorporated into the self-attention matrix. The final attention weights $P_\text {weighted}$ are obtained by combing $P_\text {attn}$, the transformed distance matrix $D'$ and the adjacency matrix $A^\text {bond}$. All $P_{weighted}$ of each head is concatenated to be processed for the following linear transformation.

2 $$\begin{aligned} \begin{aligned} P_{\text {weighted}}&= \lambda _{\text {attn}} \cdot P_{\text {attn}} + \lambda _{\text {dist}} \cdot D' + \lambda _{\text {adj}} \cdot A^\text {bond}, \\&\text {where } \, \lambda _{\text {attn}} + \lambda _{\text {dist}} + \lambda _{\text {adj}} = 1 \end{aligned} \end{aligned}$$

#### E(3)-equivariant graph convolutional layer

Following the initial processing of the molecular graph via the transformer module, each node $v_i$ is represented by an updated feature embedding that integrates attention-weighted information from its neighbors. The updated node feature $h_i^{\text {trans}}$, which integrates attention-weighted information from its neighbors, is then input into the EGNN along with the corresponding 3D spatial coordinates $c_i^{\text {3D}} \in \mathbb {R}^3$. The 3D coordinates $c_i^{\text {3D}}$ are obtained via MMFF optimization using RDKit. To integrate spatial structure in 3D space, each equivariant graph convolutional layer (EGCL) refines the molecular features while maintaining E(3)-equivariance.

The initial layer of the EGCL first inputs the relative squared distance between two coordinates $||c^{\text {3D}}_i - c^{\text {3D}}_j||^2$ along with node embeddings $h^{\text {trans}}_i, h^{\text {trans}}_j$, and edge attributes $a^\text {knn}_{ij}$. The edge attribute is derived from a k-nearest neighbor graph adjacency matrix $A^\text {knn}$ computed using Euclidean distance between atoms based on the initial atom coordinates. Message $m^1$ is aggregated from all neighboring nodes $j$ for node $i$ and is used in combination with the previous layer embedding to update the node feature $h^1$. $\phi _e$, $\phi _h$ are learned functions representing edge and node update mechanisms, respectively.

3 $$\begin{aligned} m^1_{ij}= & \phi _e \left(h^{\text {trans}}_i, h^{\text {trans}}_j, ||c^{\text {3D}}_i - c^{\text {3D}}_j||^2, a^\text {knn}_{ij}\right) \end{aligned}$$

4 $$\begin{aligned} m^1_i= & \sum _ {j \ne i} m^1_{ij}, \quad h_i^1 = \phi _h (h_i^{\text {trans}}, m^1_i) \end{aligned}$$

Additionally, in each layer the coordinate of node *i* is updated by summing weighted directional vectors from neighboring nodes, scaled by message $m_{ij}$. The scaling constant *c* controls the magnitude of positional updates. By applying equivariant transformations, the model maintains rotation and translation invariance.

5 $$\begin{aligned} c^1 = c_i^{\text {3D}} + \sum _{j \ne i} \left(c_i^{\text {3D}} - c_j^{\text {3D}}\right) \phi _x \left(m^1_{ij}\right) \end{aligned}$$

The EGCL is applied iteratively across multiple layers, with each successive layer refining both the feature embeddings $h^l$ and spatial coordinates $c^l$ while preserving E(3)-equivariance: $h^{l+1}, c ^{l+1} = \text {EGCL}(h^l, c^l, A^\text {knn})$.

For each branch, the corresponding initial node feature, adjacency matrix, distance matrix and 3D coordinate matrix are processed through the attention and equivariant graph neural network. The fragment-level graphs generated from BRICS decomposition, Murcko scaffolding, and functional group-based fragmentation are processed through an identical fragment-branch module. The representations produced by these graphs are combined using a weighted summation, expressed as:

6 $$\begin{aligned} \textbf{H}^{\text {final}}_{\text {frag}} = \lambda _b \cdot \textbf{H}^{\text {Murcko}}_{\text {frag}} + \lambda _m \cdot \textbf{H}^{\text {BRICS}}_{\text {frag}} + \lambda _f \cdot \textbf{H}^{\text {RDKit}}_{\text {frag}} \end{aligned}$$

where $\lambda _b, \lambda _m, \lambda _f$ are adjustable hyperparameters satisfying $\lambda _b + \lambda _m + \lambda _f = 1$. The final representations from the atom-level branch $\textbf{H}^{\text {final}}_{\text {atom}}$ and fragment-level branch $\textbf{H}^{\text {final}}_{\text {frag}}$ are concatenated along with Extended-Connectivity Fingerprints (ECFP). This concatenated feature vector is used as input for the prediction layer to produce toxicity scores:

7 $$\begin{aligned} \text {Prediction} = \text {Linear} \left( \text {Concat} (\textbf{H}^{\text {final}}_{\text {atom}}, \textbf{H}^{\text {final}}_{\text {frag}}, \text {ECFP})\right) . \end{aligned}$$

The model is trained using binary cross entropy loss between the predicted and target labels for classification tasks and MSE loss for regression tasks. Model parameters are updated using the gradient descent-based Adam optimizer, implemented through PyTorch python library.

#### Analysis on E(3)-equivariance

When modeling 3D molecule structures with machine learning, predictions about molecular properties should remain invariant under E(3) transformations (translations, rotations, and reflections) of the molecule’s 3D coordinates. This invariance in the final predictions is crucial for ensuring consistent and physically meaningful results. Equivariant model design enables internal representations to transform predictably under E(3) operations, maintaining the integrity of spatial features as the input molecule changes orientation or position. The combination of equivariant internal representations and invariant final predictions enables the model to generalize well and produce physically consistent (invariant) results across different molecular orientations and positions. To verify that our model maintains E(3)-equivariance, we dissect the sequential stages of processing, starting from the 2D feature graph through the transformer module to the 3D graph in the EGNN.

Initially, the transformer module processes node features based solely on atom and bond features in a 2D graph. Let $\textbf{A}$ be the adjacency matrix and $\textbf{D}$ be the distance matrix representing the connectivity of the molecule in the graph. The transformer generates updated embeddings for each node through a self-attention mechanism. Specifically, each updated node embedding $\textbf{h}_i^{\prime }$ is computed as:

$$\begin{aligned} \textbf{h}_i^{\prime } = \text {Transformer}(\textbf{h}_i, \textbf{A}, \textbf{D}). \end{aligned}$$

This step is inherently E(3)-invariant, as it does not incorporate 3D coordinate data. Consequently, the transformer module itself does not influence the model’s E(3)-equivariance, as it remains “blind” to spatial transformations.

As proven in the original paper [32], EGNN leverages pairwise distances between nodes to ensure that the output remains equivariant under E(3) transformations. As distances between points do not change under translations or rotations, it is invariant to E(3) transformations. The EGNN additionally updates each node’s embedding by aggregating information from neighboring nodes based on these distances, ensuring that spatial relationships are encoded in an equivariant manner. The updated node embedding $\textbf{h}_i^{\text {EGNN}}$ is computed as:

$$\begin{aligned} \textbf{h}_i^{\text {EGNN}} = \text {EGNN}\left( \textbf{h}_i^{\prime }, \{d_{ij}^{\text {3D}}\}_{j \in \mathcal {N}(i)}\right) , \end{aligned}$$

where $\mathcal {N}(i)$ represents the set of neighbors of node $i$.

By incorporating 3D coordinates only in this EGNN phase, our model, FATE-Tox, ensures that predictions remain E(3)-equivariant, allowing for robust and consistent analysis of molecular toxicity properties under various spatial transformations.

#### Gradient surgery for multi-task learning of toxicity

Multi-task learning (MTL) is a powerful paradigm for jointly learning multiple related tasks, enabling improved generalization through shared representations. In cheminformatics, MTL holds great promise, particularly for modeling various toxicity endpoints such as blood-brain barrier permeability (BBBP), drug-induced liver injury (DILI), skin reactions, carcinogenicity, and hERG channel inhibition. However, MTL presents unique challenges in this domain. A key issue arises from conflicting gradients during training. The diverse nature of toxicity endpoints often leads to optimization conflicts, where gradients for one task may oppose or dominate those for another. This issue is exacerbated in sharp optimization landscapes, where gradients with different magnitudes hinder convergence, causing certain tasks to dominate the optimization process. Such conflicts negatively affect model performance across tasks, as the optimization directions are misaligned, reducing the overall benefit of shared learning.

PCGrad (Projecting Conflicting Gradients), is a novel approach designed to mitigate gradient conflicts in MTL [47]. The method identifies and resolves conflicting components within task gradients after examining pairwise relationships between tasks during backpropagation. Specifically, when a conflict is detected, PCGrad modifies the gradient of one task by projecting it orthogonally to the conflicting gradient.

In our work, gradient modification using PCGrad was integrated with the Adam optimizer at the end of each training step, as follows: During each step, initial gradients are computed individually for each task, such as BBBP, DILI, and others. Pairwise dot products are then computed between gradients of all tasks in a random order. For gradient pairs exhibiting conflicts (negative dot products), the PCGrad algorithm projects each task’s gradient onto the normal plane of the gradient of the conflicting task. The modified gradients are subsequently aggregated across tasks to update the model parameters, ensuring a balanced optimization trajectory that benefits all tasks. This approach reduces destructive interference between task gradients, allowing our model to fully leverage the enriched embedding space discussed in "Ablation studies" section for improved performance across all toxicity prediction tasks.

## Results

### Experimental setup

Given the current lack of comprehensive toxicity prediction models that assess multiple toxicity endpoints simultaneously, we benchmarked a range of state-of-the-art pretrained molecular property prediction models, pretrained on large molecular datasets (e.g. ZINC20 [48]) including MolCLR [22], GraphMVP [23], MAT [25], MolFormer, and Uni-Mol [28]. In addition, we compared traditional machine learning approaches, including Random Forest (RF) [49], Support Vector Machine (SVM) [50], and Multilayer Perceptron (MLP) [51] for robust comparative analysis. Each of these models leverages different molecular representations as input, such as 2D fingerprints, 2D graphs, or 3D conformers.

We assessed model performance using the Area Under the Receiver Operating Characteristic Curve (AUROC) for each dataset, repeating the assessment three times with different seeds. For multi-task datasets-ClinTox (2 tasks) and SIDER (27 tasks)- we reported the mean AUROC averaged across all tasks. We extended our evaluation beyond single-task learning (STL) to include a multi-task learning (MTL) setup. We combined organ related single-tasked toxicity datasets by merging the training, validation, and test sets across individual datasets to perform a unified multi-task learning experiment.

### Comparison of prediction performance on seven toxicity datasets

**Table 2:** Toxicity prediction performances on seven toxicity benchmark datasets

	BBBP	DILI	Skin Rxn	Carcinogens	SIDER	ClinTox
Single-task learning						
RF	67.75 (1.04)	88.29 (1.98)	67.71 (2.07)	75.10 (3.15)	–	–
SVM	68.65 (0.00)	89.70 (0.00)	73.12 (0.02)	78.27 (0.12)	–	–
MLP	63.81 (0.55)	87.68 (0.50)	49.93 (13.81)	78.27 (0.24)	62.50 (1.11)	71.95 (1.88)
MolCLR	65.09(0.94)	81.45 (0.77)	45.05 (6.17)	74.00 (3.78)	59.87 (2.89)	82.96 (4.24)
GraphMVP	64.24 (1.27)	89.65 (0.19)	61.32 (3.50)	79.51 (4.72)	61.32 (0.71)	71.38 (1.49)
MAT	69.08 (4.68)	89.77 (0.99)	65.92 (1.13)	82.99 (3.47)	62.69 (1.54)	91.09 (0.41)
Molformer	68.60 (4.64)	88.98 (0.07)	64.28 (1.63)	73.04 (0.21)	51.41 (0.97)	71.72 (4.63)
Uni-Mol	68.76 (2.04)	88.20 (1.69)	69.48 (4.76)	82.20 (3.47)	60.23 (0.91)	91.11 (3.61)
**FATE-Tox**$_{\textbf{STL}}$	**70.15 (1.44)**	**90.53 (0.52)**	**73.33 (0.61)**	**84.16 (2.09)**	**63.29 (0.71)**	**91.37 (1.53)**
Multi-task learning						
**FATE-Tox**$_{\textbf{MTL}}$	**71.16 (1.84)**	**91.86 (0.59)**	**74.10 (0.84)**	**84.78 (0.32)**	–	–

The performances are measured in AUROC % (higher is better $\uparrow$). The mean and standard deviation of three trials for each model are provided. Additionally, we evaluate the results in a multi-task learning setting for organ-specific toxicity datasets, excluding datasets that are primarily provided as multi-task (e.g., SIDER, ClinTox). Best performances are marked in bold and second-best are underlined

Table 2 shows the experiment results of FATE-Tox and baselines under three random seeds with the best results are marked in bold. All baselines models were reproduced under the identical dataset splitting. We summarize the results as following: (1) FATE-Tox outperforms baselines on all toxicity datasets. (2) FATE-Tox is significantly better than baselines in BBBP and Carcinogens prediction tasks, with interpretable results to be discussed in "Case studies on BBBP and carcinogen compounds" section. (3) FATE-Tox enhances performance through a multi-task learning (MTL) setup, effectively augmenting complementary information that the model can naturally distinguish. The MTL setting allows the model to better capture task inter-dependencies, especially when processing fragmented datasets, and improves its ability to recognize relevant features across diverse toxicity endpoints.

Notably, during our comparative analysis, we observed that baseline models incorporating 3D conformer structural information via inter-atom distance (MAT [25], Uni-Mol [28]) showed high capacity in toxicity prediction. We believe their capability to capture stereochemical information helps explain its performance in the dataset that reflects real-world scenarios where the 3D spatial orientation of molecules are essential. Particularly, MAT effectively captured complex molecular interactions by leveraging self-attention mechanisms in majority of the datasets by capturing both local and global molecular features. In contrast, molecular fingerprint applied to traditional machine learning models, such as Support Vector Machines (SVM), which are less prone to overfitting compared to some deep learning architectures, provided stable and reliable predictions for the skin reaction tasks, indicating that key predictive features are well-represented by conventional molecular descriptors.

#### Contribution of different fragmentation methods to FATE-Tox

**Table 3:** Fragmentation weights on seven toxicity benchmark datasets

	BRICS	Bemis-Murcko Scaffolds	Functional groups (RDKit)
BBBP	**0.4**	0.3	0.3
SIDER	0.3	**0.5**	0.2
ClinTox	0.25	0.25	**0.5**
DILI	0.3	0.2	**0.4**
Carcinogens	**0.4**	0.2	**0.4**
Skin reaction	0.25	**0.5**	0.25
hERG	**0.4**	0.2	**0.4**

Each weight represents the contribution of a specific fragmentation method used to achieve the results presented in Table 2. Highest values per dataset are marked in bold

The varying importance of different fragmentation methods across toxicity datasets in Table 3 reflects the complex nature of toxicological mechanisms and the diverse chemical properties relevant to each endpoint. Each fragmentation method captures different molecular characteristics that influence their relevance to specific toxicity endpoints, as detailed below.

BRICS fragmentation produces large molecular fragments by considering the chemical context of each bond, preserving key physicochemical properties such as molecular weight, lipophilicity, and topological polar surface area (TPSA) [42]. These features are critical for endpoints like blood-brain barrier permeability (BBBP), for which global molecular characteristics, such as molecular weight, lipophilicity, and topological polar surface area (TPSA), matter more than localized sites [52]. For example, CNS-active drugs like diazepam and fluoxetine share fused-ring systems and halogenated aromatic groups, features well-retained by BRICS but potentially lost in finer scale fragmentations.

Functional group-based fragmentation (e.g., RDKit) targets specific reactive centers and toxicophores,including nitro groups, quinones, and Michael acceptors,associated with endpoints such as carcinogenicity and hepatotoxicity [1, 53–55]. These groups are well-known triggers of DNA or protein damage via electrophilic attack or oxidative stress. Compounds like acrylamide and nitrofurantoin exemplify how RDKit’s ability to extract these moieties makes it especially effective for modeling reactivity-driven toxicity.

Bemis-Murcko scaffolds focus on the core molecular framework, which is especially useful for endpoints related to structural class effects, such as skin toxicity. Their high importance in SIDER and skin reaction datasets suggests that certain scaffold types, for instance the benzene sulfonamide core in sulfamethoxazole, are predictive of dermatological adverse events, including severe conditions like Stevens-Johnson syndrome [56, 57].

Overall, the differences in highly weighted fragmentation methods across datasets likely reflect the distinct chemical properties and toxicological mechanisms relevant to each dataset. The differing granularity and specificity each method offers in identifying toxicophoric features highlights the value of combining multiple fragmentation methods for comprehensive toxicity prediction. This is also evidenced in Table 4, where the integration of all fragmentation methods outperforms individual methods for most datasets. Such multi-fragmentation integration enables the model to innately capture inter-dataset differences, allowing it to effectively handle multi-task settings across various organs with distinct chemical characteristics and toxicology profiles. Consequently, the model benefits from augmented data (combined datasets), yielding improved predictive outcomes even in multi-task setting.

### Ablation studies

**Table 4:** Ablation studies

	BBBP	SIDER	ClinTox	DILI	Skin Rxn	Carcinogens	hERG
-fragment	67.95 (2.74)	56.82 (2.17)	90.09 (0.80)	83.86 (10.34)	70.29 (2.63)	76.31 (1.86)	82.55 (1.52)
-atom	65.94 (1.47)	57.8 (5.49)	81.61 (5.84)	78.06 (0.88)	69.18 (1.69)	80.10 (1.56)	77.41 (1.90)
+BRICS	67.00 (0.26)	61.79 (0.63)	89.26 (0.50)	88.68 (2.71)	68.09 (1.16)	77.89 (0.83)	79.73 (1.93)
+Murcko-Bemis	68.44 (2.19)	61.04 (1.26)	89.65 (2.05)	86.70 (1.04)	68.75 (4.87)	80.44 (3.71)	82.82 (1.88)
+Functional Group	67.35 (1.57)	62.41 (0.78)	89.12 (0.77)	86.70 (1.04)	71.61 (3.27)	**86.64 (1.58)**	81.58 (1.74)
-molecule transformer	68.00 (0.58)	60.32 (3.79)	86.68 (1.40)	84.25 (6.07)	68.33(4.33)	80.24 (1.19)	82.31 (0.35)
**FATE-Tox** $_{\textbf{STL}}$	**70.15 (1.44)**	**63.29 (0.71)**	**91.37 (1.53)**	**90.53 (0.52)**	**73.33 (0.61)**	84.16 (2.09)	**84.30 (0.31)**

The performances are measured in AUROC %. Each entry shows the mean and standard deviation over three trials for each experimental setting. Best performances are marked in bold and second-best are underlined

#### Validation of the FATE-Tox module: performance analysis

Table 4 provides a comprehensive analysis that underscores the efficacy of our integrated approach in predicting various toxicity endpoints. The results clearly demonstrate that removing either fragment-based or atom-level features leads to a noticeable decrease in performance, which highlights the necessity of incorporating both types of features to leverage their complementary strengths. Moreover, the dual-branch architecture of our model significantly outperforms single-level graph approaches. While incorporating all three fragmentation methods scored highest in most datasets, tasks benefited with even the single use of fragmentation methods. Notably, methods that yielded the biggest increase differed across datasets.

A critical aspect of our model’s architecture is the inclusion of a molecule transformer prior to EGCLs. While the 3D conformers embedded by EGCLs are crucial, the molecule transformer’s capability to aggregate and integrate inter-node information proved to be essential across all datasets. Furthermore, to test the generalizability and the contribution of each component of the model, we evaluated the MoleculeNet-trained model on an external test set [58], which further demonstrated the robust performance of our framework and also the contributions of each component (Supplementary Table 3). These findings not only validate the architecture design choices, but also emphasize the importance of integrating multiple molecular representations and sophisticated data aggregation methods for achieving superior predictive performance.

#### FATE-Tox embedding space: multi-task learning

**Fig. 3:**
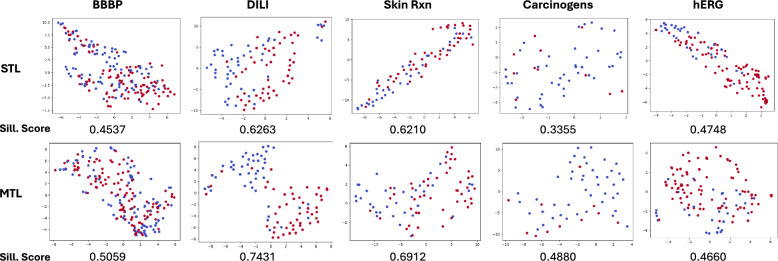
Comparison of test embedding spaces along with its silhouette scores generated by single-task learning (STL) and multi-task learning (MTL) models across the five datasets. The embeddings are projected using t-SNE and labeled based on ground truth labels (non-toxic: red / toxic: blue). Higher scores for MTL indicate superior separability and cohesion in the shared embedding space, illustrated in the learnt embedding spaces visualized using t-SNE

In a multi-task learning setting, the learned embeddings are shared across all tasks, capturing representations that balance both task-specific and task-agnostic features. This space is enriched by cross-task knowledge transfer, enabling it to capture broader and potentially more robust patterns in the data. To evaluate the quality of these embeddings, we performed a clustering-based analysis by applying binary k-means clustering and computing silhouette scores for the resulting cluster labels. This analysis focuses on the intrinsic structure of the shared MTL embedding space and its ability to produce distinct and meaningful clusters for a given task.

We compared the concatenated embedding from the final layer of each atom and fragment branch in the corresponding STL/MTL setting. The results, summarized in Fig. 3, show that MTL embeddings consistently achieved higher silhouette scores across most datasets. This indicates that the shared MTL feature space not only capture patterns that generalize across toxicity tasks but also produces embeddings that are inherently better at distinguishing between task-specific categories. The effect is also visually illustrated for the test dataset of each toxicity, comparing the embedding spaces learned in STL and MTL setting. The embeddings are projected into a two-dimensional space using t-SNE visualization, and the colors represent ground truth labels. The MTL embeddings generally exhibit more well-separated clusters, aligning with the higher silhouette scores reported.

This analysis underscores the strength of the MTL approach, not only for task performance but also for generating embeddings with higher intrinsic quality. These findings align with the hypothesis that shared representations in MTL facilitate richer feature learning by leveraging synergies across tasks.

#### FATE-Tox embedding space: multi-view approach

**Fig. 4:**
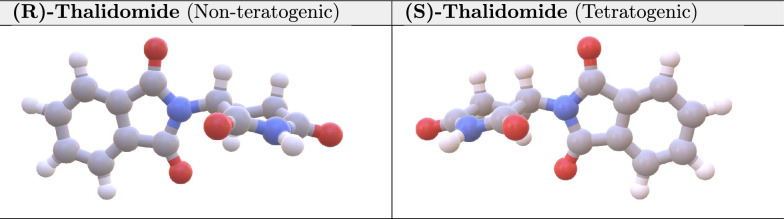
3D conformer of (R)/(S)-Thalidomide

In our study, we further demonstrate the effectiveness of our multi-view approach by visualizing the embedding spaces of ($R$)-thalidomide and ($S$)-thalidomide. The two molecules are enantiomers, which are mirror images of each other. Enantiomers have the same molecular formula and the same connectivity between atoms but differ in the spatial arrangement around a chiral center. Hence the distinction between ($R$)- and ($S$)-thalidomide becomes apparent only in 3D representations as presented in Fig. 4. Nonetheless, such spatial differences exhibit profound biological differences, with ($R$)-thalidomide being non-teratogenic and ($S$)-thalidomide known for its high teratogenicity [59, 60]. This distinction highlights the limitations of using only 2D molecular representations in toxicity prediction, as key stereochemical differences are often lost, and may lead to inaccurate risk assessments.

**Fig. 5:**
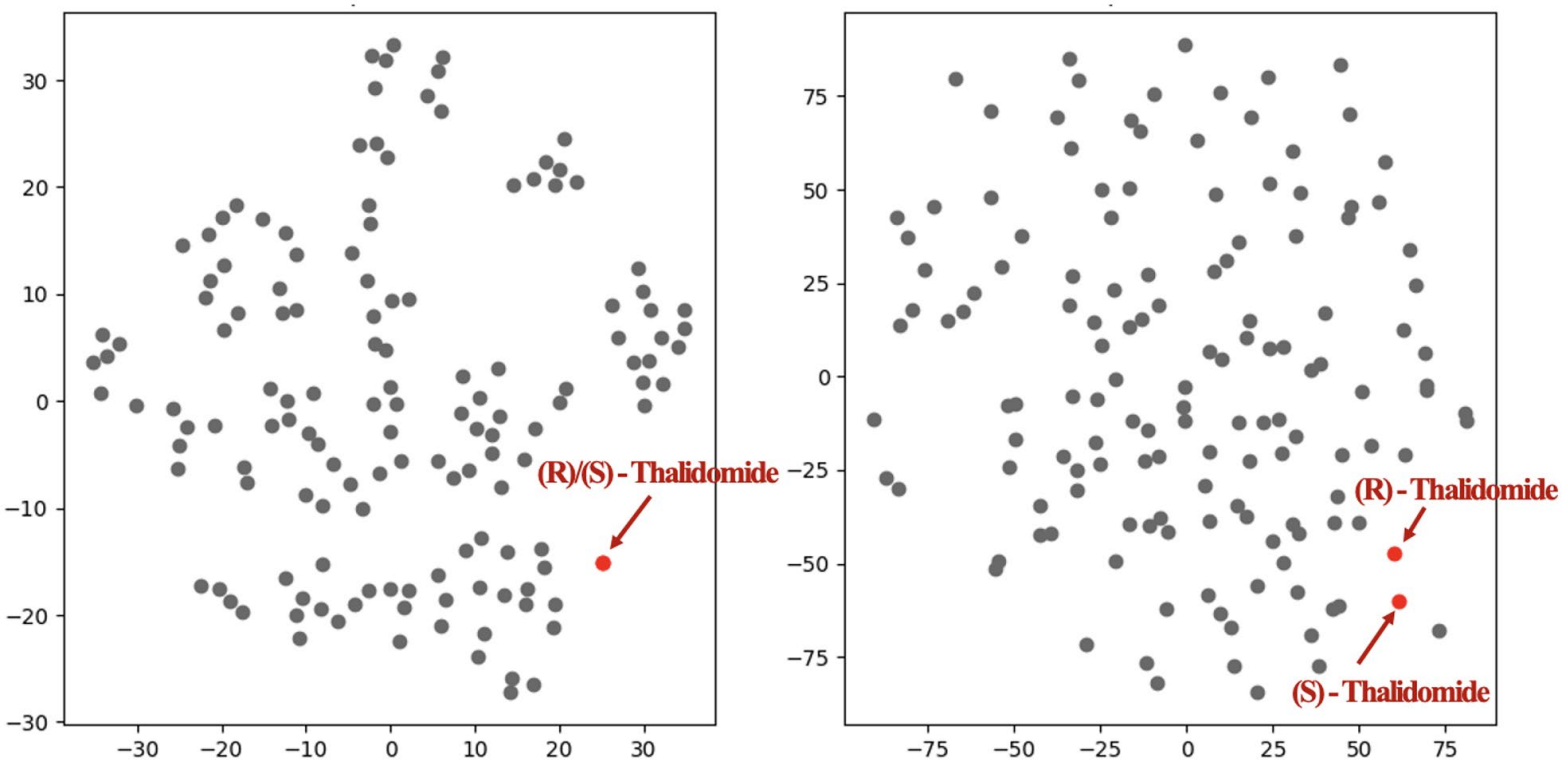
t-SNE visualization of (R/S)-Thalidomide embeddings. (Left) MAT (2D-only), (Right) FATE-Tox (2D+3D) approach. The red points represent Thalidomide, while the remaining points correspond to the test dataset of SIDER

To showcase such properties, we visualized the embedding spaces of two methods: 2D embeddings of MAT, and 3D incorporated embeddings from FATE-Tox, with t-SNE dimensionality reduction (Fig. 5). The embeddings were retrieved by the pretrained models of each for the SIDER dataset. By plotting the test dataset of SIDER, $R$/$S$ isomers were not distinguishable under MAT embeddings. In contrast, our multi-view embeddings with 3D conformer incorporation, separated these isomers, capturing stereoisomer-specific toxicity risks. Nonetheless, 2D embeddings may focus on planar or topological molecular properties, providing complementary geometric information. This validates that multi-view representations significantly improve the model’s capacity to identify and differentiate between subtle but consequential molecular differences in chemical compounds.

### Case studies on BBBP and carcinogen compounds

To illustrate the interpretability of the proposed FATE-Tox, we conducted an in-depth analysis on the Blood-Brain Barrier (BBB) penetration and carcinogens dataset by visualizing multi-head averaged attention coefficients. Specifically, attention scores from the final layer of the molecule transformer were employed to identify substructures important for toxicity prediction. Atoms with higher attention coefficients were illustrated with deeper intensities of red. For edges that link atoms of high coefficients than the average coefficient within the molecule, the edges were colored gray. Furthermore, we have compared the interpretability of our attention coefficients with existing eXplainable AI (XAI) frameworks including SHapley Additive exPlanations (SHAP) [61] and Grad-CAM [62] analyses, detailed in Supplementary Table 2.

#### Blood brain barrier penetration of anti-histamines and beta-blockers

**Table 5:**
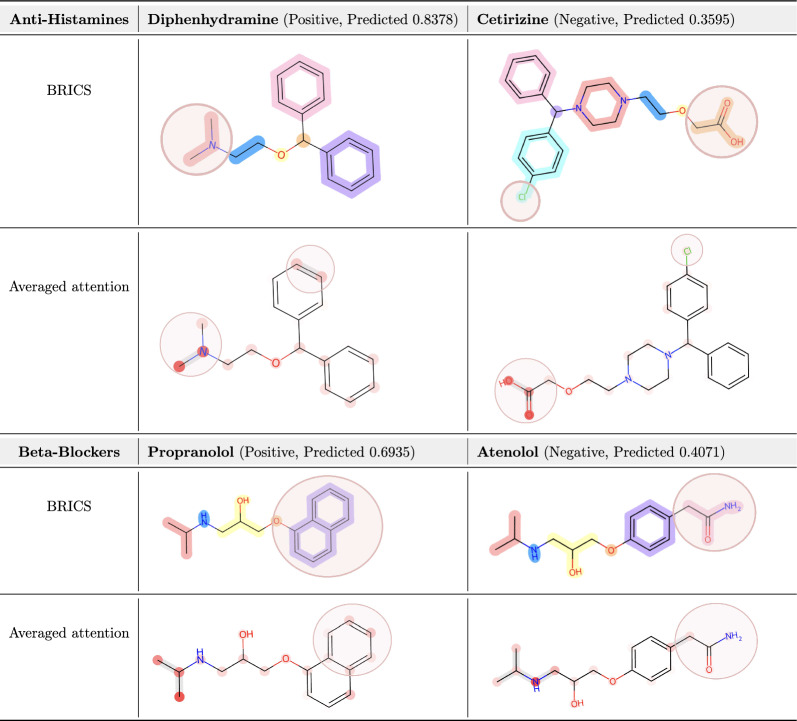
Identification of important substructures in anti-histamines and beta-blockers. Averaged attention coefficients are visualized, with the molecule’s corresponding BRICS decomposition

Diphenhydramine and propranolol are widely known for their capabilities of penetrating the BBB, resulting in adverse effects on the central nervous system. Complementing their inconveniencies, cetirizine and atenolol has been developed to minimize the penetration of BBB and thus minimize CNS side-effects [63–65]. Table 5 shows that for both anti-histamines and beta-blockers, the attention visualizations highlighted key distinct substructures that significantly contributed to the overall polarity of the compounds. Among molecules of high structural similarity (same drug class), FATE-Tox effectively captured characteristic substructures contributing to lower blood-brain barrier permeability within the atom-level graph. We provide the molecular mechanisms based on differing structures of the molecules, contributing to the difference in ability to cross the blood-brain barrier. This reduction is associated with decreased central nervous system side effects, such as sedation, drowsiness, fatigue observed in cetirizine and atenolol compared to diphenhydramine and propranolol.

Specifically, diphenhydramine consists of a tertiary amine attached to two hydrophobic benzene rings. The absence of polar functional groups makes diphenhydramine highly lipophilic (LogP 3.3$-$3.6), allowing it to dissolve in the lipid bilayer of the BBB and pass via passive diffusion [66, 67]. On the other hand, cetirizine has a carboxyl group and a chloride substituent, making it significantly more polar than diphenhydramine (logP 0.3$-$0.6). This leads to minimal passive diffusion across the BBB. Additionally, the carboxyl group is deprotonated in our body of pH 7.4, making cetirizine negatively charged to further prevent BBB passage. Likewise, propranolol is highly lipophilic (LogP 3.0$-$3.5) driven by a naphthalene (benzene-based) ring. Atenolol contains a hydrophilic amide group, significantly reducing lipophilicity (LogP 0.16). FATE-Tox successfully captures all the mentioned key substructures leading to difference in polarity and BBB penetration, demonstrating FATE-Tox’s capability to pinpoint regions relevant to specific pharmacokinetic properties.

The dual-branch architecture of FATE-Tox, which integrates atom-level and BRICS-fragmented graphs, proved instrumental in achieving accurate predictions. The high weighting of the BRICS fragmentation for the BBBP dataset graph (Table 3), resulted in atom-level attention coefficients appearing in a fragmented pattern corresponding to these substructures. This highlighted the model’s proficiency in learning and representing potential toxicophores through guidance from the fragment branch. Such congruence underscores FATE-Tox’s utility in formulating hypotheses for potential 3D toxicophores.

#### Carcinogenicity of Sudan I

**Fig. 6:**
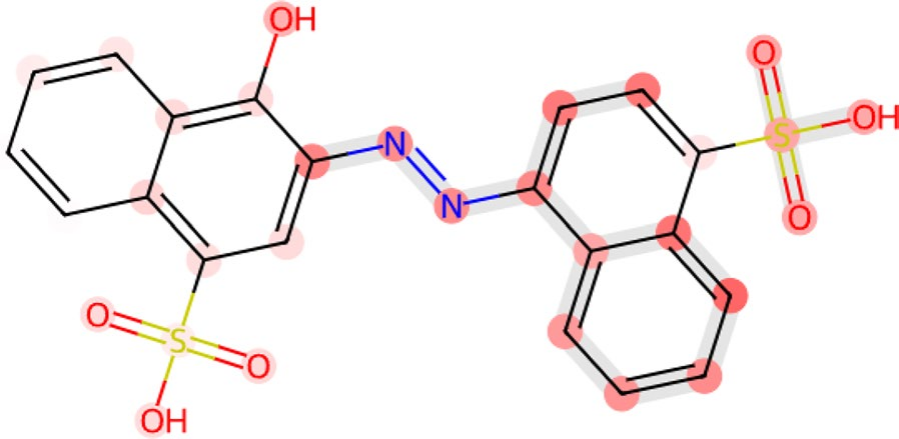
Visualization of FATE-Tox averaged attention coefficients of Sudan I, a synthetic azo dye with potential carcinogenicity

Sudan I exhibits high carcinogenic potential due to its aromatic azo group (-N=N-R), which, releases DNA-binding aromatic amines that induce mutations. In detail, the azo bond is known to be bio-reduced by azoreductases to yield primary aromatic amines, further metabolized by cytochrome P450 to form nitrenium ions that react with guanine residues in DNA. Such reaction process results in DNA adducts and causes mutagenesis. In addition, its electrophilic sulfonic acid groups increase mutagenic risk through reactivity with cellular macromolecules. Sulfonic acid groups also inhibit conjugation reactions crucial for detoxification pathways, leading to prolonged retention of carcinogenic intermediates and increased risk of DNA damage [68–70]. Metabolic processing, particularly in the liver, further transforms Sudan I into reactive carcinogenic byproducts, leading to DNA damage and potential cancer initiation. FATE-Tox accurately predicts Sudan I in the test set as highly carcinogenic (probability: 0.9899) by capturing these key toxicophores and identifying critical substructures in 3D space, including the azo and sulfonic acid groups (Fig. 6). Through interpretable visualizations of attention coefficients, FATE-Tox highlights these regions with high importance, aligning with literature on carcinogenic mechanisms and enhancing predictive reliability for toxicity assessment.

## Conclusion and future works

In this study, we presented a novel toxicity prediction framework leveraging a dual-branch architecture that integrates atom-level and multiple fragment-level features. By combining graph-based transformers with E(3)-equivariant graph neural networks, our approach effectively captures the spatial dependencies and chemical properties necessary for accurate toxicity predictions. Our model demonstrated the ability to discriminate between enantiomers, which are separable only in 3D space, and identify potential 3D toxicophores in alignment with literature through attention visualization. The implementation of multiple fragmentation methods, each of which exhibits varying levels of importance depending on the dataset, enables the model to capture both generalizable and task-specific patterns. This flexible featurization strategy, combined with spatial and relational processing, enhances the model’s ability to provide comprehensive predictions of toxicity endpoints, especially within the multi-task learning setting. Incorporation of PCGrad maximizes the benefits of our setting by removing conflicting components to ensure balanced optimization, thereby improving prediction performance in multi-task learning setting.

While our model achieved promising results, future research could explore adaptive fragmentation strategies that dynamically adjust to the chemical context, further improving the interpretability and predictive performance of the model. Additionally, extending the model to account for time-dependent toxicity patterns (e.g., bioaccumulation or metabolic pathways) could provide a more comprehensive understanding of long-term chemical effects. By addressing these areas, we envision developing a comprehensive, interpretable, and scalable system for toxicity prediction that bridges the gap between in-silico modeling and real-world applications.

## Supplementary Information

Supplementary material 1.

## Data Availability

The source code for FATE-Tox is available at https://github.com/sumin124/FATE-Tox. The data used in this study is publicly available from the official dataset repositories of MoleculeNet and Therapeutics Data Commons (TDC).
