# Supplementary material for: Fate-tox: fragment attention transformer for E(3)-equivariant multi-organ toxicity prediction
Source: J Cheminform. 2025 May 14;17:74. doi: 10.1186/s13321-025-01012-5 (PMC12080013; doi:10.1186/s13321-025-01012-5)
Supplement: Supplementary file 1 — Supplementary material 1. [file 13321_2025_1012_MOESM1_ESM.pdf]

SUPPLEMENTARY INFORMATION FOR: FATE-Tox: Fragment Attention Transformer for E(3)-Equivariant Multi-Organ Toxicity Prediction

Sumin Ha[1,†], Dongmin Bang[2,3,†], and Sun Kim[1,2,3,4,*]

[1]Interdisciplinary Program in Artificial Intelligence, Seoul National University, Seoul, Republic of Korea
[2]Interdisciplinary Program in Bioinformatics, Seoul National University, Seoul, Republic of Korea
[3]AIGENDRUG Co., Ltd., Seoul, Republic of Korea
[4]Department of Computer Science and Engineering, Seoul National University, Seoul, Republic of Korea
[†]These authors contributed equally to this work.
[*]For whom the correspondence should be: sunkim.bioinfo@snu.ac.kr

Contents

1 Supplementary Tables **2**
1. Statistical Evaluation of Performance Table 1 2
2. Comparison of FATE-Tox's Attention Scores with Explainable Machine Learning Approaches 3
3. Performance of FATE-Tox and Ablation Checkpoints on an External Blood-Brain Barrier Dataset (B3DB) 4

2 Supplementary Methods **5**
1. Datasets 5
2. Node Attributes for Graph Construction 5

References **5**

1 Supplementary Tables

Supplementary Table 1. Statistical Evaluation of Performance Table 1

Statistical evaluation of Table 1 with one-sided paired T-test. p-values of each models' performances compared with the highest performing model (FATE-Tox) are provided.

	**BBBP**	**DILI**	**Skin Rxn**	**Carcinogens**	**SIDER**	**ClinTox**
FP-RF	0.025	0.065	0.019	0.012	-	-
FP-SVM	0.004	0.016	0.005	0.004	-	-
FP-MLP	0.002	0.011	0.045	0.005	0.002	0.264
MolCLR	0.001	0.003	0.007	0.011	0.059	0.099
GraphMVP	0.008	0.008	0.013	0.066	0.002	0.064
MAT	0.245	0.010	0.002	0.128	0.353	0.272
Molformer	0.221	0.007	0.004	0.002	0.004	0.000
Uni-Mol	0.097	0.036	0.114	0.100	0.468	0.004
FATE-Tox	Best	Best	Best	Best	Best	Best

Supplementary Table 2. Comparison of FATE-Tox's Attention Scores with Explainable Machine Learning Approaches

Comparison of FATE-Tox's attention scores compared to explainable machine learning approaches (e.g., SHAP[1], Grad-CAM[2]) on case study compounds of the main manuscript.

	SHAP	Grad-CAM	FATE-Tox
Diphenhydramine	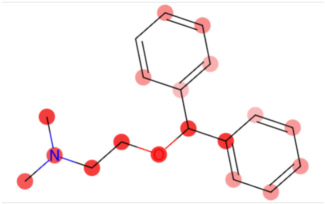	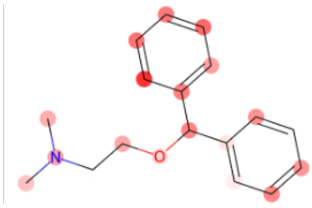	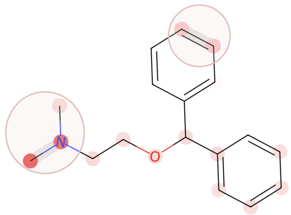
Cetirizine	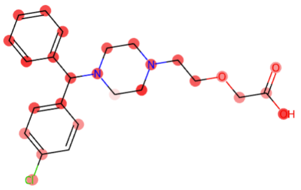	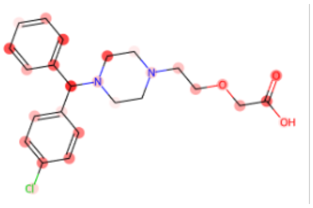	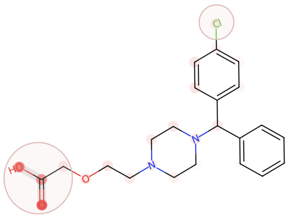
Propranolol	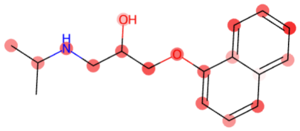	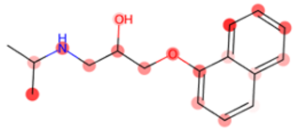	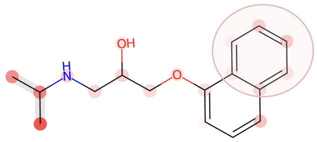
Atenolol	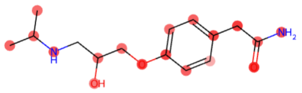	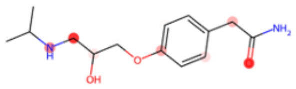	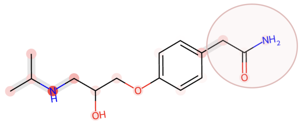
Sudan Id	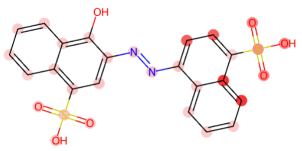	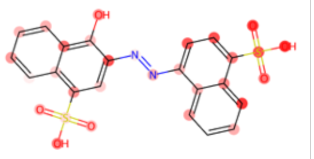	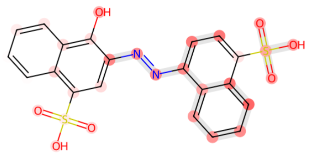

Supplementary Table 3. Performance of FATE-Tox and Ablation Checkpoints on an External Blood-Brain Barrier Dataset (B3DB)

Evaluation of generalizability on external BBBP dataset (B3DB[3]), measured using accuracy, AUROC, recall, precision, F1-Score. Performance of FATE-Tox and Ablation Checkpoints on External Blood-Brain Barrier Dataset Across Five Metrics.

Model	Accuracy	AUROC	Precision	Recall	F1-Score
− fragment	0.6373	0.7595	0.7909	0.5494	0.6484
− atom	0.6455	0.7759	0.8226	0.5324	0.6465
+ BRICS	0.7175	0.7950	0.7985	0.7167	0.7554
+ Murcko-Bemis	0.7533	0.8084	0.7447	0.9050	0.8171
+ Functional Group	0.7094	0.7951	0.7980	0.6998	0.7457
**FATE-Tox**	**0.7872**	**0.8374**	**0.8061**	**0.8565**	**0.8305**

2 Supplementary Methods

Datasets

This section presents a comprehensive overview of the seven datasets utilized for toxicity prediction in this study. These datasets are categorized based on specific toxicity endpoints, encompassing both human clinical data and in vivo experimental results. By incorporating organ-specific as well as general toxicity datasets, this study aims to ensure a robust and comprehensive evaluation of toxicity prediction models.

- **Clinical Endpoint Datasets** provide toxicity data derived from human trials and post-marketing studies, capturing adverse outcomes that directly impact drug safety assessments.
  - **ClinTox** dataset includes a collection of drugs with documented clinical toxicity profiles, allowing for predictions aligned with real-world outcomes.
  - **DILI (Drug-Induced Liver Injury)** dataset compiles information on drugs and compounds associated with liver toxicity, an important criterion for drug approval.
  - **SIDER (Side Effect Resource)**: dataset catalogs information on drug-related adverse effects from public documents and package inserts, providing a broad spectrum of adverse event data. This resource supports toxicity predictions relevant to both common and rare drug-induced side effects, aiding the development of models that reflect a drug's full toxicity profile.
- **In Vivo Toxicity Datasets** capture data from experimental animal studies conducted in preclinical stages.
  - **Skin Reaction** dataset contains information on dermatological reactions.
  - **Carcinogens** dataset provides data essential for predicting carcinogenic risk.
  - **BBBP (Blood-Brain Barrier Permeability)** dataset assesses compounds' ability to cross the blood-brain barrier, providing a basis for neurotoxicity predictions.
  - **hERG (Human Ether-à-go-go-Related Gene)** dataset includes compounds with known interactions with the hERG potassium channel, where blockages are associated with cardiac arrhythmias. hERG toxicity predictions are essential for cardiovascular safety evaluations.

Node Attributes for Graph Construction

This section describes the node features used for constructing the atom graph in our model. Each atom in a molecular graph is characterized by a set of features to encode its chemical properties effectively. Table 6.1 provides an overview of these features. These features collectively serve as inputs to the graph-based model, enabling it to capture the molecular structure and chemical properties accurately.

Index	Features
0-11	Atom Identity (One-hot vector)
12-17	Number of heavy neighbors (One-hot vector)
18-22	Number of hydrogen atoms (One-hot vector)
23-25	Formal Charge (One-hot vector)
26-29	Hybridization (One-hot vector)
30	Is Cyclic
31	Is Aromatic
32	Bond Order

Table 1. Node Features for Graph Construction

References

1. Lundberg, S. M. & Lee, S.-I. A unified approach to interpreting model predictions. *Adv. neural information processing systems* **30** (2017).
2. Selvaraju, R. R. *et al.* Grad-cam: Visual explanations from deep networks via gradient-based localization. In *Proceedings of the IEEE international conference on computer vision*, 618–626 (2017).
3. Meng, F., Xi, Y., Huang, J. & Ayers, P. W. A curated diverse molecular database of blood-brain barrier permeability with chemical descriptors. *Sci. Data* **8**, DOI: 10.1038/s41597-021-01069-5 (2021).
